# Validating the Simplified Endoscopic Mucosal Assessment for Crohn’s Disease: A Novel Method for Assessing Disease Activity

**DOI:** 10.1093/ibd/izac183

**Published:** 2022-09-01

**Authors:** Jeremy Adler, Richard B Colletti, Lenore Noonan, Tyler M Berzin, Adam S Cheifetz, Laurie S Conklin, Timothy C Hoops, Christopher S Huang, Blair Lewis, Daniel S Mishkin, Kim Hung Lo, Yongling Xiao, Sheri Volger

**Affiliations:** *C.S. Mott Children’s Hospital, Michigan Medicine, University of Michigan, Ann Arbor, MI, USA; Susan B. Meister Child Health Evaluation and Research Center, Michigan Medicine, University of Michigan, Ann Arbor, MI, USA; University of Vermont Children’s Hospital, Burlington, VT, USA; Janssen Research & Development, LLC, Spring House, PA, USA; Beth Israel Deaconess Medical Center, Division of Gastroenterology, Harvard Medical School, Boston, MA, USA; Beth Israel Deaconess Medical Center, Division of Gastroenterology, Harvard Medical School, Boston, MA, USA; Janssen Research & Development, LLC, Spring House, PA, USA; **Immunology Global Medical Affairs, Janssen Pharmaceutical Companies (a subsidiary of Johnson & Johnson), Horsham, PA, USA; Boston Medical Center, Section of Gastroenterology, Boston University School of Medicine, Boston, MA, USA; Division of Gastroenterology, Mount Sinai Health System, New York, NY, USA; Atrius Health, Division of Gastroenterology, Boston, MA, USA; Janssen Research & Development, LLC, Spring House, PA, USA; Cytel, Inc., Waltham, MA, USA; Janssen Research & Development, LLC, Spring House, PA, USA

**Keywords:** validation, SEMA-CD, SES-CD, endoscopic disease activity, Crohn’s disease

## Abstract

**Background:**

To demonstrate treatment efficacy in Crohn’s disease (CD), regulatory authorities require that trials include an endoscopic remission/response end point; however, standardized endoscopic assessment of disease activity, such as the Simple Endoscopic Score for Crohn’s Disease (SES-CD), is not typically recorded by clinicians in practice or outside of clinical trials. The novel Simplified Endoscopic Mucosal Assessment for Crohn’s Disease (SEMA-CD) was developed to be easy to use in routine clinical practice and as a trial end point. We conducted a study to assess and validate the reliability and feasibility of SEMA-CD as a measure of endoscopic disease activity.

**Methods:**

Pre- and post-treatment ileocolonoscopy videos of pediatric (*n* = 36) and adult (*n* = 74) CD patients from 2 ustekinumab clinical trials were each scored with SEMA-CD by 2 to 3 professional central readers, blinded to clinical history and other video scorings; the correlation between SEMA-CD and SES-CD previously completed during the trials was assessed. Sensitivity to change, inter- and intrarater reliability, and comparative ease of scoring were also assessed.

**Results:**

The SEMA-CD strongly correlated with SES-CD (Spearman *ρ* = 0.89; 95% confidence interval, 0.86-0.92). Pre- to post-treatment changes in SEMA-CD vs in SES-CD were strongly correlated, and the correlation remained strong between the scores when compared by study population (pediatric, adult), disease severity, and video quality. Intra- and inter-rater reliability were good, and SEMA-CD was rated easier than SES-CD to score 63.0% of the time, although slightly more difficult than SES-CD to score <1.0% of the time.

**Conclusions:**

The SEMA-CD is reliable, reproducible, sensitive to change, and easy to use in both pediatric and adult patients with CD.

## Graphical Abstract

**Graphical Abstract:**
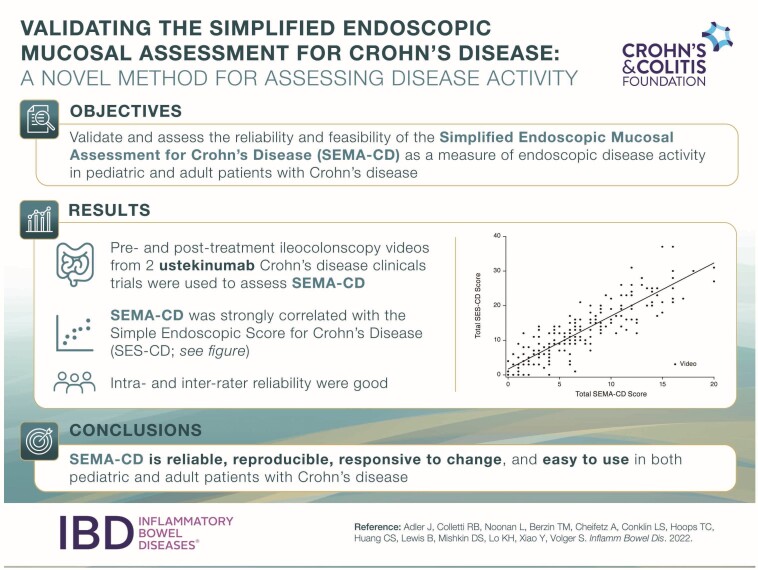

**Key Messages**

**What is already known?**

Although the Simple Endoscopic Score for Crohn’s Disease (SES-CD) is often used to assess endoscopic disease activity and severity in clinical trials, it can be too complex and time-consuming for clinicians to use in everyday practice.

**What is new here?**

The novel Simplified Endoscopic Mucosal Assessment for Crohn’s Disease (SEMA-CD) provides an easy but accurate method for clinicians to quantify disease activity and severity in real-world practice.

**How can this study help patient care?**

Patient care would benefit from both simplifying and standardizing how colonoscopy results are reported.

## Introduction

United States Food and Drug Administration (FDA) and European Medicines Agency (EMA) guidance encourages evaluating the reliability and relevance of real-world data and the methodologies used to generate real-world evidence from registry-based studies supporting the benefit-risk evaluation of new medical products.^1,2^ For evaluating new treatments for Crohn’s disease (CD), the FDA and EMA both recommend conducting clinical trials that evaluate mucosal healing and include an endoscopic remission/response end point, which is typically derived from endoscopic videos, as an objective measure of disease activity for demonstrating CD treatment efficacy.^3,4^

Registries collect data on signs, symptoms, laboratory tests, and clinical outcomes. Endoscopy data are challenging and typically too difficult to collect routinely.^5^ The qualitative nature of these endoscopic findings further limits the use of registry data for evaluating the endoscopic end point of mucosal healing, which is considered the gold standard for assessing CD treatment efficacy.^6^

To utilize registry data adequately to assess mucosal disease activity in CD, a simple, standardized method is needed to quantify ileocolonoscopy findings. Although the Simple Endoscopic Score for Crohn’s Disease (SES-CD) is often used to assess endoscopic disease activity and severity in clinical trials,^7^ it can be too complex and time-consuming for clinicians to use in everyday practice.^5^ Patient care would benefit from both simplifying and standardizing how colonoscopy results are reported. The novel Simplified Endoscopic Mucosal Assessment for Crohn’s Disease (SEMA-CD) was developed to provide an easy but accurate method for clinicians to quantify disease activity and severity in real-world practice.^8^ In a pilot study,^8^ SEMA-CD was found to be accurate, reproducible, and correlate strongly with the validated SES-CD.^7^ Nevertheless, further testing of the measurement properties and external validity of SEMA-CD is needed.

We designed and conducted a study to assess the validity, reliability, usability, and feasibility of using SEMA-CD vs SES-CD as a clinical measure (end point) of endoscopic disease activity in both pediatric and adult populations. Ileocolonoscopy videos performed as part of 2 clinical trials were evaluated to validate the prospective use of SEMA-CD for determining mucosal activity across a range of disease severity. We hypothesized that the assessment of mucosal activity measured with SEMA-CD would be positively associated with the assessment of mucosal activity measured with SES-CD.

## Materials and Methods

### Study Design

We conducted a retrospective study utilizing ileocolonoscopy videos and SES-CD from 2 ustekinumab (STELARA; Janssen Biotech, Inc., Horsham, PA, USA) clinical trials: the UNISTAR phase 1, randomized, double-blind, pharmacokinetic study of ustekinumab in pediatric patients with moderately to severely active CD^9^ (ClinicalTrials.gov; NCT02968108); and the SEAVUE phase 3b, multicenter, randomized, blinded, active-controlled study comparing the efficacy and safety of ustekinumab vs adalimumab in the treatment of biologic-naïve adult patients with moderately to severely active CD^10^ (ClinicalTrials.gov; NCT03464136). The ileocolonoscopy videos were scored using SEMA-CD, and the results were compared with the SES-CD previously obtained during these clinical trials.

### Study Patients

In the UNISTAR study, pediatric patients with moderately to severely active CD received ustekinumab intravenous induction followed by subcutaneous ustekinumab maintenance.^9^ Disease severity was categorized using the Pediatric Crohn’s Disease Activity Index (PCDAI).^11^ Within 2 weeks prior to baseline, patients underwent an ileocolonoscopy and were randomized 1:1 to 2 different dosing regimens of ustekinumab. At week 16, patients underwent a second ileocolonoscopy.

In the SEAVUE study, adult patients (18 years and older) with moderately to severely active CD were randomized 1:1 to ustekinumab or adalimumab for 1 year.^10^ Prior to randomization, all patients underwent an ileocolonoscopy; inclusion criteria required the presence of at least 1 ulcer of any size within the ileum or colon. Disease severity was categorized using the Crohn’s Disease Activity Index (CDAI).^12^ At week 52, patients underwent a second ileocolonoscopy.

In both the UNISTAR and SEAVUE studies, each of the pre- and post-treatment ileocolonoscopy videos were scored with the SES-CD by blinded central readers.

The current study was conducted in accordance with Good Clinical Practice and Health Insurance Portability and Accountability Act of 1996 requirements. Only de-identified data were used in the study, and the confidentiality of the patient records was maintained at all times.

### Study Objectives

The primary objective was to assess the correlation between SEMA-CD and SES-CD derived from the same ileocolonoscopy videos representing a wide range of mucosal disease severity in pediatric and adult patients with CD. Secondary objectives were to assess the correlation between SEMA-CD and SES-CD across disease severity; the inter-rater and intrarater reliability of the SEMA-CD; the correlation between the change in SEMA-CD and change in SES-CD from baseline scores (SEMA-CD sensitivity to change in disease activity pre- and post-treatment); the usability measured by the ease of scoring SEMA-CD compared with SES-CD; and the feasibility of applying the SEMA-CD.

### Study Procedures

The ileocolonoscopy videos from the UNISTAR and SEAVUE studies were de-identified (eg, all patient demographics, clinical history, and identifiers were redacted) prior to entry in the video display system for the study readers. All patients from UNISTAR and a random sample of patients from SEAVUE were included in this study. Only patients with an available pre-treatment ileocolonoscopy video were included in the study data set.

From the pool of professional central readers (Bioclinica, Princeton, NJ, USA) who had reviewed and scored the videos in the UNISTAR and SEAVUE studies using SES-CD, 4 central readers were trained to score de-identified ileocolonoscopy videos using SEMA-CD. Each video was reviewed in random order by 2 readers (central reader 1 and central reader 2), with the score of a third reader (central adjudication reader 3) used for adjudication, if necessary. If there was a disagreement between central reader 1 and central reader 2 of >5 points (ie, if the scores were not within the maximum difference allowed) using SEMA-CD, the video was allocated to the central adjudication reader 3 to assign an adjudication score, completing the review. The central adjudication reader 3 assessed the ileocolonoscopy videos under the same conditions as central reader 1 and central reader 2. All the central readers were blinded to the scores of the other central readers and to the previously scored SES-CD.

As previously reported by Adler et al,^8^ the SEMA-CD is scored by assigning a numerical value ranging from 0 (endoscopic remission) to 4 (severe disease) for each bowel region (ileum and colon; Table 1). The overall colon is scored as a whole based on the most severe colonic segment. The number of colonic segments with any degree of active disease is recorded, regardless of the severity of individual segments. The overall colon score is then multiplied by the number of involved colonic segments, and the result is added to the ileum score. In contrast to SES-CD,^7^ strictures of the ileocecal valve are to be considered in the ileum score, whereas a nonintubation of the ileum without stricture is considered to be “not assessed.” The SEMA-CD scores range from 0 to 20, where 0 indicates inactive CD activity (endoscopic remission) and 20 indicates the worst disease severity. Clinically meaningful cutoffs for SEMA-CD have not been established. For the purpose of this study, a priori, we proposed the following candidate cutoff scores: 0, inactive; 1, minimal; 2 to 4, mild; 5 to 9, moderate; and ≥10, severe. These cutoffs were selected based on the range of observed SEMA-CD and SES-CD scores computed in a prior study.^8^

**Table 1.** Scoring of the Simplified Endoscopic Mucosal Assessment for Crohn’s Disease (SEMA-CD).^8^

Points	Ileum	Colon	Description	
0	◯ Endoscopic remission	◯ Endoscopic remission	Normal	
1	◯ Minimal disease	◯ Minimal disease	No more than a few aphthous ulcers Otherwise normal	
2	◯ Mild disease	◯ Mild disease	Scattered aphthous ulcers or small ulcers No large ulcers (ie, no ulcers >2 cm) Narrowing, ok if it can be passed	
3	◯ Moderate disease	◯ Moderate disease	Scattered large ulcers or widespread small ulcers Multiple passable stenoses No nonpassable stricture or visible fistulas	
4	◯ Severe disease	◯ Severe disease	Widespread large ulcers Non passable stricture or visible fistula	
‒	◯ Not assessed	◯ Not assessed		
**Colonic segments involved**	⧠ Ascending	⧠ Transverse	⧠ Descending	⧠ Rectum

Strictures of the ileocecal valve should be considered in the ileum score. Nonintubation of ileum without stricture should be considered “not assessed.”

### Study Outcomes

Validity^13,14^ was measured by assessing the strength of the overall correlation between SEMA-CD and SES-CD and the strength of the correlation between SEMA-CD and SES-CD across categories of disease severity. To assess the sensitivity of SEMA-CD to change in disease activity pre- and post-treatment, the correlation between the change from pre- to post-treatment in SEMA-CD and change from pre- to post-treatment in SES-CD was measured.

Reliability^13,14^ of the SEMA-CD was assessed by measuring inter- and intrarater reliability: agreement between multiple central readers rating a given video, and agreement between a central reader rating a given video twice. To assess intrarater reliability, 15% of the cases were reassigned to the original reader after a minimum of 2 weeks had passed to minimize the possibility of recall bias.

To determine the usability (ease of scoring) of SEMA-CD compared with SES-CD, readers were asked to rate the ease of scoring SEMA-CD using the following scale: much easier than SES-CD, somewhat easier than SES-CD, slightly easier than SES-CD, neither easier nor more difficult than SES-CD, slightly more difficult than SES-CD, somewhat more difficult than SES-CD, or much more difficult than SES-CD.

Feasibility was assessed by measuring the proportion of videos that were not scorable by SEMA-CD and the correlation between SEMA-CD and SES-CD by video quality category (as rated by the SEMA-CD readers: “readable, but not optimal” and “optimal”).

### Statistical Analyses

Baseline characteristics of the study patients were summarized using descriptive statistics. In general, continuous variables were summarized using descriptive statistics (eg, mean, standard deviation, median, and range), and categorical variables were summarized using counts and percentages. Missing data were not imputed.

The primary analysis assessed the correlation between SEMA-CD and SES-CD for the same patient video using the full data set of adult and pediatric videos. For SEMA-CD, the median score of all readings was used given that each video was scored by 2 (without adjudication) or 3 readers (with adjudication). Spearman rank-order correlation coefficient (*ρ*) and the associated 95% confidence interval (CI) were computed. A Spearman *ρ* of 0.6 was specified as a meaningful threshold.

The secondary analyses assessed the correlation between SEMA-CD and SES-CD across categories of disease severity using Spearman *ρ* for the combined pediatric and adult patients. Founded on the baseline SES-CD, disease severity was defined as inactive (0 to 2), mild (3 to 6), moderate (7 to 15), or severe (≥16).^15^ To determine the sensitivity of SEMA-CD to change in disease activity pre- and post-treatment, the correlation between the change from pre- to post-treatment in SEMA-CD and change from pre- to post-treatment in SES-CD was assessed using Spearman *ρ* for the combined patients.

Descriptive statistics of SEMA-CD were reported by initial read and reread by the same reader, and the distribution was presented graphically. Difference in SEMA-CD between initial read and reread across all the readers was reported as a weighted average of mean difference of each reader across all the readers. Intraclass correlation coefficient (ICC) and associated 95% CI were estimated using a linear mixed effect model to assess intrarater agreement among the SEMA-CD reported for an initial read and reread by the same reader. Based on the 95% CI for the ICC, values <0.50 were considered poor reliability, from 0.50 to 0.75 were considered moderate reliability, from 0.75 to 0.90 were considered good reliability, and >0.90 were considered excellent reliability.^16^ To assess inter-rater reliability, descriptive statistics of SEMA-CD were reported by reader (central reader 1 and central reader 2, in fixed order) by pair of readers, and the distribution was presented graphically. Difference in SEMA-CD from all reader pairs was reported as the inverse-variance-weighted average of the stratum-level mean differences assuming the same order of readers in each pair. Intraclass correlation coefficient and the associated 95% CI were estimated using a linear mixed effect model to assess the inter-rater agreement among the entire reading pool for SEMA-CD. Inter-rater reliability of the categorical SEMA-CD cutoffs was also assessed by computing weighted Cohen kappa statistic (95% CI) for 6 paired combinations of the 4 central readers. Kappa values from 0.41 to 0.60 were considered moderate agreement, from 0.61 to 0.80 were considered good agreement, and >0.80 were considered very good agreement.^17^

Usability was described by summarizing the 7-point ratings. Feasibility was assessed by analyzing the correlation between SEMA-CD and SES-CD by video quality category (“readable but not optimal” and “optimal”) using Spearman *ρ* for the combined patients, and by reporting the number and percentage of nonscorable videos.

## Results

### Patient Characteristics

In total, 110 patients were included in the analyses (36 pediatric patients from UNISTAR and 74 adult patients from SEAVUE). Most of the pediatric patients were White (86.1%), more than half were female (63.9%), and three-quarters were ≥12 to <18 years in age (75.0%; Table 2). Most of the adult patients were White (87.8%), more than half were female (51.4%), and approximately three-quarters were ≥18 to <45 years in age (71.6%). The median (range) age at initial CD diagnosis was 8.9 years (1-16 years) for the pediatric patients and 29.9 years (9-67 years) for the adult patients. The median (range) CD duration was 3.8 years (1-12 years) for the pediatric patients and 3.4 years (0-40 years) for the adult patients. More than half of the pediatric patients (60.0%) and adult patients (58.9%) had both ileal and colonic involvement. At baseline, the median (range) PCDAI score was 42.5 (18-65), and the median (range) CDAI score was 289.5 (213-463). The overall median (range) SES-CD was 13 (0-37). According to baseline SES-CD endoscopic disease severity, 40.9% had moderate disease, and 35.5% had severe disease. The median (range) C-reactive protein (CRP) was 8.1 mg/L (0-232 mg/L).

**Table 2.** Patient characteristics.

	UNISTAR Phase 1 Study	SEAVUE Phase 3b Study	Total
**Analysis set**			
N	36	74	110
**Age (years)**			
Mean (SD)	13.2 (2.76)	37.1 (12.69)	29.3 (15.43)
Median (range)	13.0 (6-17)	36.5 (18-69)	26.0 (6-69)
≥2 to <6	0	0	0
≥6 to <12	9 (25.0%)	0	9 (8.2%)
≥12 to <18	27 (75.0%)	0	27 (24.5%)
≥18 to <45	0	53 (71.6%)	53 (48.2%)
≥45 to <65	0	19 (25.7%)	19 (17.3%)
≥65	0	2 (2.7%)	2 (1.8%)
**Sex**			
Female	23 (63.9%)	38 (51.4%)	61 (55.5%)
Male	13 (36.1%)	36 (48.6%)	49 (44.5%)
**Race**			
Asian	0	7 (9.5%)	7 (6.4%)
Black or African American	0	1(1.4%)	1 (0.9%)
White	31 (86.1%)	65 (87.8%)	96 (87.3%)
Other	1 (2.8%)	0	1 (0.9%)
Multiple	1 (2.8%)	1 (1.4%)	2 (1.8%)
Not reported	2 (5.6%)	0	2 (1.8%)
Unknown	1 (2.8%)	0	1 (0.9%)
**Weight (kg)**			
Mean (SD)	41.2 (11.39)	72.6 (22.52)	62.3 (24.51)
Median (range)	41.1 (20-66)	68.4 (42-149)	57 (20-149)
<40	17 (47.2%)	0	17 (15.5%)
≥40 to ≤ 55	16 (44.4%)	17 (23.0%)	33 (30.0%)
>55 to ≤ 85	3 (8.3%)	41 (55.4%)	44 (40.0%)
>85	0	16 (21.6%)	16 (14.5%)
**Age at initial CD diagnosis (years)**			
Mean (SD)	8.8 (3.85)	30.5 (11.92)	23.4 (14.30)
Median (range)	8.9 (1-16)	29.9 (9-67)	21.5 (1-67)
**CD duration (years)**			
Mean (SD)	4.4 (2.83)	6.7 (8.35)	5.9 (7.10)
Median (range)	3.8 (1-12)	3.4 (0-40)	3.6 (0-40)
**Involved gastrointestinal areas**			
N	35	73	108
Ileum only	2 (5.7%)	15 (20.5%)	17 (15.7%)
Colon only	12 (34.3%)	15 (20.5%)	27 (25.0%)
Ileum and colon	21 (60.0%)	43 (58.9%)	64 (59.3%)
**PCDAI score**			
N	35	0	35
Mean (SD)	43.7 (10.56)	0	43.7 (10.56)
Median (range)	42.5 (18-65)	0	42.5 (18-65)
**CDAI score**			
N	0	74	74
Mean (SD)	0	300.7 (59.23)	300.7 (59.23)
Median (range)	0	289.5 (213-463)	289.5 (213-463)
**SES-CD score**			
Mean (SD)	15.6 (10.22)	12.5 (7.34)	13.5 (8.47)
Median (range)	14 (0-37)	12 (0-27)	13 (0-37)
**Endoscopic disease severity per SES-CD**			
Inactive (0-2)	4 (11.1%)	1 (1.4%)	5 (4.5%)
Mild (3-6)	4 (11.1%)	17 (23.0%)	21 (19.1%)
Moderate (7-15)	12 (33.3%)	33 (44.6%)	45 (40.9%)
Severe (≥16)	16 (44.4%)	23 (31.1%)	39 (35.5%)
**CRP, mg/L**			
Mean (SD)	20.3 (25.92)	16.6 (31.29)	17.8 (29.57)
Median (range)	11.4 (0-94)	6.6 (0-232)	8.1 (0-232)

Abbreviations: CD, Crohn’s disease; CDAI, Crohn’s Disease Activity Index; CRP, C-reactive protein; N, number of patients; PCDAI, Pediatric Crohn’s Disease Activity Index; SD, standard deviation; SES-CD, Simplified Endoscopic Activity Score for Crohn’s Disease.

Ns are provided for each parameter with missing values.

### Validity

#### SEMA-CD and SES-CD correlation

**Figure 1.**
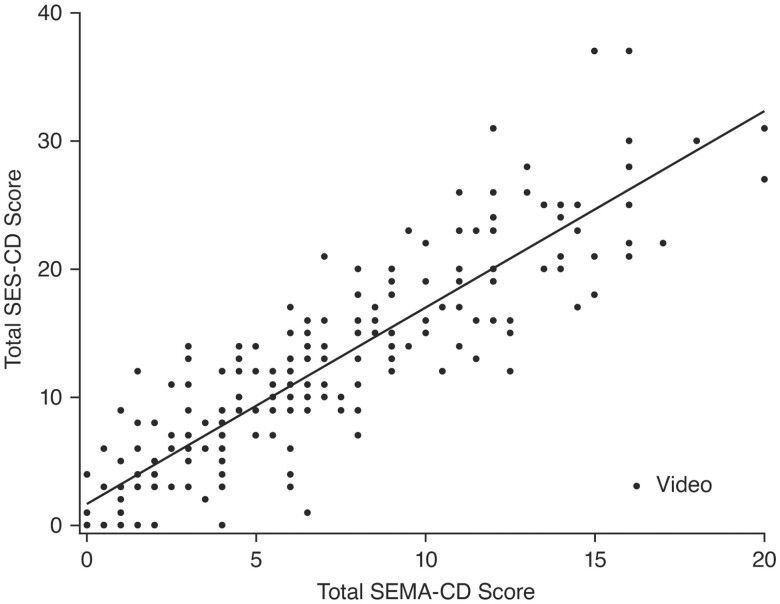
Scatter plot demonstrating the correlation of SEMA-CD with SES-CD (*ρ* = 0.89; 95% CI, 0.86-0.92). Abbreviations: CI, confidence interval; SEMA-CD, Simplified Endoscopic Mucosal Assessment for Crohn’s Disease; SES-CD, Simplified Endoscopic Activity Score for Crohn’s Disease. Each point in the figure corresponds to a video scored using SEMA-CD and SES-CD. For SEMA-CD, each video was scored by 2 readers with a third reader included for adjudication as needed. The median SEMA-CD of all readings for each video was used in the analysis.

Of the 110 patients, 218 videos were scored by 2 central readers (or 3 readers, if adjudicated) using SEMA-CD, and 219 videos were previously scored using SES-CD. The median (range) SEMA-CD was 5.5 (0.0-20.0), and the median (range) SES-CD was 10.0 (0.0-37.0). Overall, SEMA-CD correlated strongly with SES-CD (*ρ =* 0.89; 95% CI, 0.86-0.92; Figure 1). By clinical trial, the correlation remained strong between scores when comparing SEMA-CD and SES-CD in pediatric (UNISTAR study; *ρ* = 0.94; 95% CI, 0.90-0.96) and adult (SEAVUE study; *ρ* = 0.86; 95% CI, 0.80-0.89) patients.

#### SEMA-CD and SES-CD correlation across disease severity

When analyzed by disease severity, SEMA-CD strongly correlated with SES-CD (all Spearman *ρ* ≥ 0.6). The Spearman *ρ* (95% CI) was 0.85 (0.48-0.96) for patients with inactive disease (*n* = 5), 0.69 (0.49-0.82) for those with mild disease (*n* = 21), 0.72 (0.60-0.81) for those with moderate disease (*n* = 45), and 0.83 (0.75-0.89) for those with severe disease (*n* = 39).

#### SEMA-CD sensitivity to change in disease activity pre- and post-treatment

**Figure 2.**
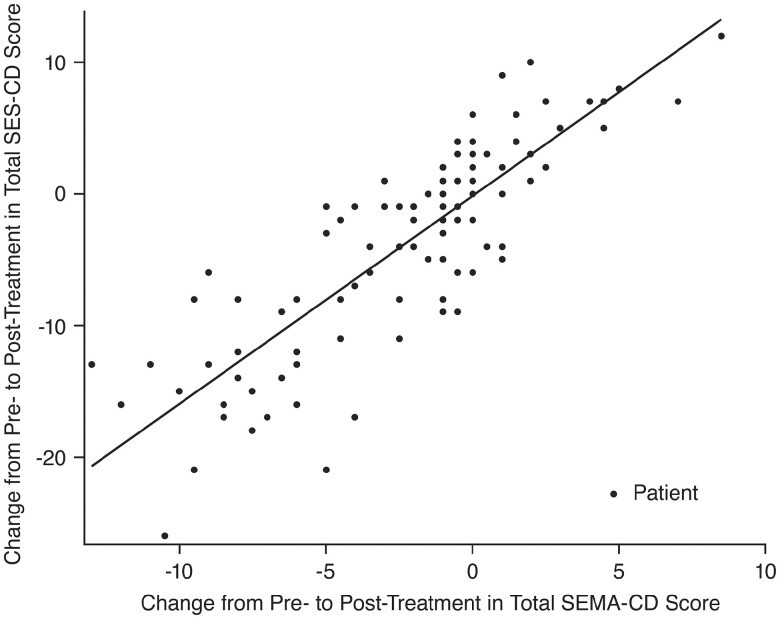
Scatter plot demonstrating the correlation of change from pre- to post-treatment in SEMA-CD and change from pre- to post-treatment in SES-CD (*ρ* = 0.84; 95% CI, 0.77-0.89). Abbreviations: CI, confidence interval; SEMA-CD, Simplified Endoscopic Mucosal Assessment for Crohn’s Disease; SES-CD, Simplified Endoscopic Activity Score for Crohn’s Disease. Each point in the figure corresponds to change in scores from pre- to post-treatment that was measured using SEMA-CD and SES-CD for each patient. For SEMA-CD, each video was scored by 2 readers with a third reader included for adjudication as needed. The median SEMA-CD of all readings for each video was used in the analysis.

The median (range) change in score from pre- to post-treatment was −1.0 (−13.0 to 8.5) for SEMA-CD and −2.0 (−26.0 to 12.0) for SES-CD. Overall, relative to the change from pre- to post-treatment, SEMA-CD strongly correlated with SES-CD (*ρ* = 0.84; 95% CI, 0.77-0.89; Figure 2).

### Reliability

#### Inter-rater reliability

The overall mean (95% CI) difference was −0.25 (−0.52 to 0.02) for the SEMA-CD for the 6 paired combinations of 4 central readers in the reader pool. The inter-rater agreement for SEMA-CD among the pairs of readers was good, with an ICC (95% CI) of 0.89 (0.85-0.91). The inter-rater agreement for SEMA-CD on the categorical scale was moderate to very good, with kappa ranging from 0.59 to 0.86 across the reader pairs. Overall, the inter-rater agreement for SEMA-CD on the categorical scale was good, with an overall kappa (95% CI) of 0.75 (0.69-0.81).

#### Intrarater Reliability

The central readers reread up to 17 videos each, at least 22 days following each initial read date. The overall mean (95% CI) difference between initial read and reread was −0.07 (−0.40 to 0.25) for the intrarater agreement for the SEMA-CD. The intrarater agreement for SEMA-CD for the initial read and reread by the same reader was good, with an ICC (95% CI) of 0.93 (0.88-0.96).

### Usability

**Figure 3.**
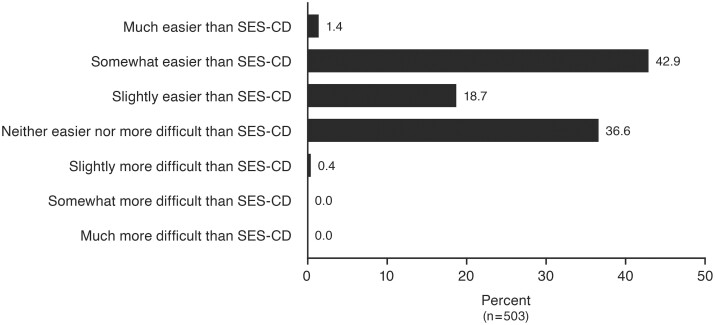
Usability of SEMA-CD vs SES-CD by number of videos scored (percent). Abbreviations: *n*, number of videos; SEMA-CD, Simplified Endoscopic Mucosal Assessment for Crohn’s Disease; SES-CD, Simplified Endoscopic Activity Score for Crohn’s Disease.

The SEMA-CD scoring was easier than SES-CD scoring. For 503 video readings, the central readers rated SEMA-CD as follows: slightly, somewhat, or much easier than SES-CD to score 63.0% of the time; equal to SES-CD to score 36.6% of the time; and slightly more difficult to score 0.4% of the time (Figure 3).

### Feasibility

All the pre- and post-treatment videos were scorable for video quality using SEMA-CD (*n* = 218) and SES-CD (*n* = 219). For the video quality category “readable, but not optimal,” the proportion was 51.8% for SEMA-CD and 51.6% for SES-CD. For the video quality category “optimal,” the proportion was 48.2% for SEMA-CD and 48.4% for SES-CD. Again, SEMA-CD strongly correlated with SES-CD across video quality (for “readable, but not optimal,” *ρ* = 0.88, 95% CI, 0.83-0.91; and for “optimal,” *ρ* = 0.90, 95% CI, 0.86-0.93).

## Discussion

Overall, we demonstrated that SEMA-CD strongly correlated with SES-CD and was sensitive to change in disease activity, with pre- to post-treatment changes in SEMA-CD strongly correlating with pre- to post-treatment changes in SES-CD. In addition, the correlation remained strong between SEMA-CD and SES-CD when comparing scores in pediatric and adult patients across SES-CD disease severity, and SEMA-CD was rated easier than SES-CD to score 63% of the time, and the same as or easier than SES-CD to score 99.6% of the time.

Our study is the first to assess the validity and reliability of the SEMA-CD in both pediatric and adult patients with CD and to measure the sensitivity of SEMA-CD to the change in disease activity in individual patients following treatment in the controlled environment of clinical trials. In addition to demonstrating statistically significant results, our study tested a priori cutoffs for SEMA-CD, furthering the ability of clinical practitioners and researchers to understand the scores and identify clinically meaningful treatment benefits. Our study has limitations, however. The same central readers did not score both the SEMA-CD and SES-CD at the same time because the SES-CD from the clinical trials was utilized. Nonetheless, these central readers belonged to the same pool of trained, professional central readers (Bioclinica, Princeton, NJ, USA) who conducted the reviewing and scoring for both our study and the clinical trials using standardized procedures. The SES-CD requires over 20 entries to tabulate the total score, rendering it difficult to complete by endoscopists who are not used to or who do not routinely use this scoring system.^5^ The central readers have considerable experience with scoring the SES-CD, whereas this was their first experience with scoring the SEMA-CD; with more experience, scoring the SEMA-CD may improve over time. In addition, experience as a central reader may have the potential to introduce bias, as it may be easier for an experienced central reader to score colonoscopy results and to achieve rater agreement than for a less-experienced endoscopist in clinical practice; however, this may not be relevant since, in the prior pilot study of SEMA-CD, the practicing pediatric gastroenterologists, none of whom were professional central readers, had excellent inter-rater agreement.^8^

The strong correlations that we observed in our study are consistent with the findings of 2 previous cross-sectional studies. One study demonstrated that SEMA-CD was strongly correlated with SES-CD derived from endoscopy videos.^8^ A second study showed that SEMA-CD applied retrospectively to endoscopy reports from electronic medical records is strongly correlated with video-derived SEMA-CD and SES-CD.^18^

In a study of the ileocolonoscopy videos of 57 pediatric patients from a single practice, a strong correlation was found between SEMA-CD and SES-CD when assessed blindly by central readers (*ρ* = 0.98; *P* < 0.0001) who reported scoring the SEMA-CD to be substantially easier.^8^ The inter-rater reliability, measured with weighted Cohen kappa statistic, was 0.80 for SEMA-CD and 0.86 for SES-CD (values for kappa >0.80 were considered very good). The intrarater reliability, measured in the same manner as the inter-rater reliability, was 0.83 for SEMA-CD and 0.90 for SES-CD.

A subsequent study of these same pediatric patients (*n* = 57) found that the retrospective application of SEMA-CD for evaluating colonoscopy reports strongly correlated with the SES-CD of the ileocolonoscopy videos.^18^ This thus demonstrates that the SEMA-CD could be accurately and reliably applied to the evaluation of endoscopic disease severity, as documented in electronic health records containing colonoscopy reports. As in the previous study, the inter- and intrarater reliability for the SEMA-CD was very good.

Advancements in CD treatment rely on traditional clinical trials to generate evidence to support regulatory approvals of medical products and to inform patient care.^1,2,19^ The FDA and EMA both require documentation of validated and standardized assessment of mucosal activity as an objective measure of disease activity in these trials.^3,4^ Clinical trial participants with CD are not fully representative of real-world patient populations,^20^ limiting the applicability of trial findings to real-world clinical situations. Inflammatory bowel disease (IBD) registries, on the contrary, intentionally collect patient demographic and treatment data, store and monitor health status and disease activity, and even evaluate the quality and delivery of the patients’ care.^21^ As a result, the real-world data that these registries collect are potentially of great value. Validation of the SEMA-CD is important for regulatory decision-making that depends not only on the reliability and relevance of real-world data but also on the standardized methods used to generate real-world evidence of mucosal disease activity and severity.

## Conclusion

In both pediatric and adult patients with CD, SEMA-CD is a valid and reliable method for measuring mucosal disease activity from ileocolonoscopy videos collected during clinical trials. The SEMA-CD strongly correlates with SES-CD and is easier to use, providing researchers with a simple and standardized alternative to SES-CD. Furthermore, SEMA-CD may be a valuable method for assessing both clinically meaningful improvements in mucosal disease activity and the effectiveness of CD treatments. Future studies should examine the generalizability of SEMA-CD in community practice and in real-world data collected by IBD registries.^18,21^

## Data Availability

The data sharing policy of Janssen Pharmaceutical Companies of Johnson & Johnson is available at https://www.janssen.com/clinical-trials/transparency. As noted on this site, requests for access to the study data can be submitted through the Yale Open Data Access (YODA) Project site at http://yoda.yale.edu.
